# Advanced Characterization Techniques and Theoretical Calculation for Single Atom Catalysts in Fenton-like Chemistry

**DOI:** 10.3390/molecules29163719

**Published:** 2024-08-06

**Authors:** Zhaokun Xiong, Zhicheng Pan, Zelin Wu, Bingkun Huang, Bo Lai, Wen Liu

**Affiliations:** 1 The Key Laboratory of Water and Sediment Sciences, College of Environmental Sciences and Engineering, Peking University, Ministry of Education, Beijing 100871, China; scuxzk@scu.edu.cn; 2 Sichuan Province Engineering Technology Research Center of Water Safety and Water Pollution Control, Haitian Water Group, Chengdu 610065, China; 3 Sino-German Centre for Water and Health Research, Sichuan University, Chengdu 610065, China; wuzelinscu@163.com (Z.W.); hbkscu@163.com (B.H.); laibo@scu.edu.cn (B.L.)

**Keywords:** single-atom catalysts, environmental remediation, peroxymonosulfate, structural analysis, density functional theory

## Abstract

Single-atom catalysts (SACs) have attracted extensive attention due to their unique catalytic properties and wide range of applications. Advanced characterization techniques, such as energy-dispersive X-ray spectroscopy, X-ray photoelectron spectroscopy, transmission electron microscopy, scanning electron microscopy, and X-ray absorption fine-structure spectroscopy, have been used to investigate the elemental compositions, structural morphologies, and chemical bonding states of SACs in detail, aiming at unraveling the catalytic mechanism. Meanwhile, theoretical calculations, such as quantum chemical calculations and kinetic simulations, were used to predict the catalytic reaction pathways, active sites, and reaction kinetic behaviors of SACs, providing theoretical guidance for the design and optimization of SACs. This review overviews advanced characterization techniques and theoretical calculations for SACs in Fenton-like chemistry. Moreover, this work highlights the importance of advanced characterization techniques and theoretical calculations in the study of SACs and provides perspectives on the potential applications of SACs in the field of environmental remediation and the challenges of practical engineering.

## 1. Introduction

In recent years, Fenton-like reactions that catalyze persulfate (peroxymonosulfate (PMS) and peroxydisulfate (PDS)) and other oxidants (peroxyacetic acid (PAA) and periodate (PI)) to produce reactive oxygen species (ROS) such as radicals or nonradicals to achieve efficient degradation of organic pollutants molecules have received widespread attention [1,2,3,4,5,6]. The focus of Fenton-like reactions is the design of superior catalysts to improve the activation efficiency and catalytic reaction kinetics. In homogeneous catalysis, the reactants, catalysts, and products are all in the same aqueous phase and have extensive contact areas, making it possible to obtain high catalytic efficiency with less catalyst addition [7,8,9,10]. However, the problem exists that homogeneous catalysts cannot be recovered after reaction, resulting in waste and, possibly, the pollution of water environments. Heterogeneous catalysts have the advantages of easy separation after reaction and recyclable use. However, the traditional heterogeneous catalysts with metal nanoparticles as the leading active site have poor catalytic performance due to the low utilization rate of metal atoms and the inability to expose the internal areas. Single-atom catalysts (SACs) are a class of materials that anchor a single metal atom to support [11,12,13,14,15]. The research on SACs has determined that dispersing metal particles at the atomic level maximizes the use of metal atoms and improves the selectivity of catalytic reactions [16,17,18,19,20]. Metals and coordination atoms are responsible for generating active sites in heterogeneous catalysis, similar to enzymes or homogeneous catalysts [21,22,23]. This utilization of metal atoms gives SACs an excellent performance and reduces the consumption of precious metals to produce low-cost catalysts [24,25]. Because of their unique advantages, SACs are widely used in energy and environmental applications, such as oxygen reduction reaction (ORR), carbon dioxide reduction reaction (CO_2_RR), oxygen evolution reaction (OER), and hydrogen evolution reaction (HER), and used as rechargeable battery electrodes [26,27,28,29,30,31,32]. However, the review of the application of SACs in Fenton-like chemistry is limited.

Determining the existence of isolated single atoms and their spatial distribution is the key to developing superior SACs [33,34,35,36,37]. Currently, the characterization methods of SACs are mainly divided into electron microscopy and spectroscopy, among which the spectroscopy mainly includes X-ray absorption near edge spectroscopy (XANES) and extended X-ray absorption fine structure spectroscopy (EXAFS), infrared spectroscopy and nuclear magnetic resonance (NMR) [38,39,40]. The most straightforward and convincing approach is to use electron microscopy techniques such as scanning tunneling microscope (STM) and atomic spherical correction electron microscopy (ACEM) to directly image individual atoms dispersed on the surface of a carrier with a high specific surface area. Moreover, XANES and EXAFS spectroscopy can also provide information on the dispersion and oxidation states of single atoms. In addition, by using suitable probe molecules (such as CO, NH_3_, and pyridine), infrared spectroscopy can also be used to assess the presence of individual metal atoms and, to a certain extent, quantify the percentage of individual metal atoms dispersed on the surface of the carrier [41,42]. In addition, the changes of the SACs in the catalytic reaction process, the proof of the actual active site, and the elucidation of the catalytic reaction mechanism have not been deeply understood [43,44]. The analysis of single atomic catalytic reaction mechanisms using theoretical calculation has been widely used [45,46]. Therefore, it is urgent to systematically summarize the advanced characterization methods and the theoretical calculation analysis of SACs reported so far.

In this work, the advanced characterization methods of SACs developed in recent years are summarized in detail, including electron microscopy and spectroscopy, and the characteristics, advantages, and disadvantages of each characterization method are analyzed. In addition, based on the work of SACs in Fenton-like chemistry, the current application direction of theoretical calculation is summarized systematically. The unique mechanism of SACs in catalysis is discussed, and theoretical guidance is provided for determining the real active center in the catalysis process and improving the catalytic performance of SACs through atomic-level regulation. Finally, the main problems facing the development of SACs are claimed, and the research challenges and prospects in single-atom catalysis are expounded.

## 2. Advanced Characterizations

**Figure 1:**
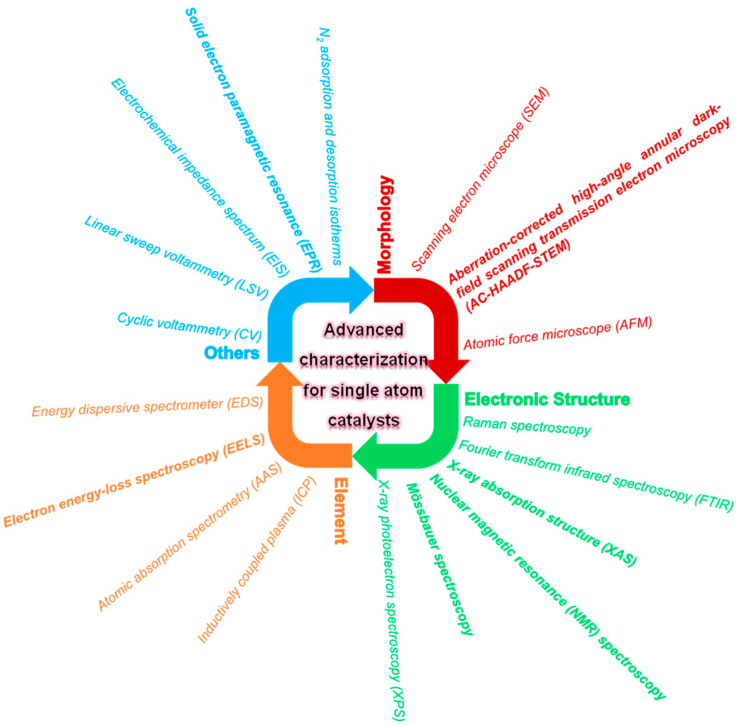
Categorization of the common SACs characterization methods.

In order to further study the electronic states and metal–carrier interactions of SACs, a variety of advanced characterization techniques are widely used. These techniques study the microscopic environment from different perspectives, which can deepen the understanding of the mechanism of SACs. Different characterization tools give respective information on the morphologies, structures, compositions, and pore properties. Until now, various emerging techniques, including aberration-corrected high-angle annular dark field scanning transmission electron microscopy (HAADF-STEM), X-ray absorption fine structure (XAFS) measurement, infrared (IR) and NMR spectroscopy techniques, Raman, solid electron paramagnetic resonance (EPR), and Mössbauer spectroscopy, have been developed to investigate the detailed microstructure of SACs [14,15,47,48,49,50]. Given that a single technique usually cannot give comprehensive information, the complementary results are adopted using different tools to identify the SACs better and understand their comprehensive properties. The common SAC characterization methods applied in persulfates activation are categorized in Figure 1. Nowadays, advanced characterization tools used for identifying the morphologies, structures, and compositions of SACs include aberration-corrected HAADF-STEM, EXAFS, and XANES. Moreover, the electrochemical properties, such as reducibility and conductivity of SACs, can be investigated by electrochemical techniques, including linear sweep voltammetry (LSV), cyclic voltammetry (CV), and electrochemical impedance spectrum (EIS). Also, the concentrations of PMS/PDS during the reaction should be determined to measure the utilization of PMS/PDS by catalysts. In recent years, the theoretical calculation based on DFT has offered deep insight into the active sites and explored the reaction mechanism. It is worth noting that the combined various characterization methods enable us to understand the composite structures of SACs comprehensively. The objects and tools for the emerging characterization of SACs applied in persulfates activation are summarized, but not limited to, those listed below.

### 2.1. Electron Microscopy

**Figure 2:**
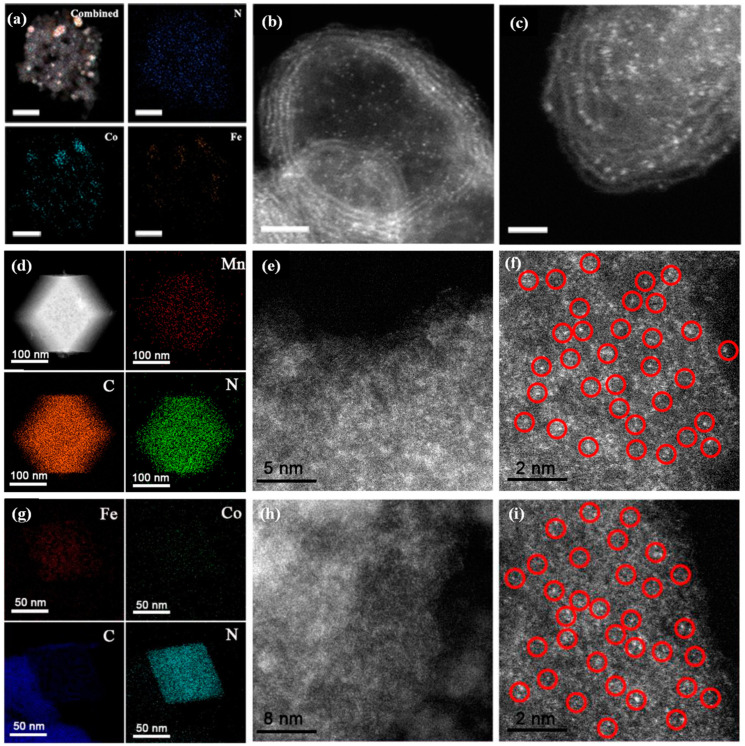
(**a**) EDX mappings and (**b**,**c**) HAADF-STEM images of FeCo-NC-2. Scale bars: (**a**) 50 nm, (**b**) 2 nm, and (**c**) 1 nm. Reproduced with permission [56]. Copyright 2018, American Chemical Society. (**d**) Element mapping images and (**e**,**f**) HAADF-STEM images of the Mn-ISAs@CN, red circles represent individual dispersed metal atoms. Reproduced with permission [57]. Copyright 2020, Elsevier. (**g**) Element mapping images, and (**h**,**i**) HAADF-STEM images of the FeCo@NC-1, red circles represent individual dispersed metal atoms. Reproduced with permission [58]. Copyright 2020, Elsevier, The Netherlands.

The catalytic properties of SACs are bound up with their microstructures and compositions. Transmission electron microscopy (TEM) is the most commonly used microscope to elucidate the morphology, size, dispersity, and distribution of SACs. In some cases, even the crystal structure and porosity of the SACs could be identified by TEM [51,52,53]. To determine the size and morphology of SACs with a very tiny degree, various tools, such as high-resolution TEM (HR-TEM), HAADF-STEM, and selected area electron diffraction (SAED), are utilized complementarily to explain the crystallinity and also identify the phase based on the lattice fringe [54]. Based on the principle of Rutherford scattering, the image intensity collected from HAADF-STEM is proportional to the square of the atomic number for the selected element, which allows heavy atoms to show bright contrast compared with light atoms [55]. This tool can be readily applied to characterize the dispersity and distribution of metal elements in SACs. Li et al. clearly observed the dispersion of single Co/Fe atoms anchored on the porous N-doped graphene (FeCo-NC) derived from a Fe-Co Prussian blue analogue (PBA) by using aberration-corrected HAADF-STEM (Figure 2a–c) [56]. It can be observed that the light spots of single Co/Fe atoms were well dispersed across the entire graphene spheres. Similarly, a single Mn atom anchored on N-doped porous carbon (Mn-ISAs@CN) derived from Mn(acac)_3_@ZIF-8 was fabricated by Yang et al. [57]. The EDX mapping images (Figure 2d) revealed that the Mn, C, and N were distributed homogeneously throughout the structure. The HAADF-STEM images (Figure 2e,f) show that single Mn atoms are homogeneously dispersed in the N-doped porous carbon, which can be clearly observed by the isolated bright dots marked with red cycles (Figure 2f). In addition, Yang et al. also synthesized isolated diatomic Fe-Co anchored on N-doped porous carbon (FeCo@NC) based on Fe(acac)_3_/Co(acac)_2_@ZIF-8 [58]. HAADF-STEM and EDX mapping images also illustrated the homogeneous dispersion of Fe-Co single atoms (Figure 2g–i). The electron microscope image is intuitive and enables the observation of the morphology, particle size, and distribution of the catalyst, which facilitates our understanding of the microstructure of the catalyst. However, the field of view of the electron microscope is relatively limited, and usually only a localized area of the sample can be observed. Therefore, combined analyses with other characterizations tools may be required when a comprehensive understanding of the sample properties is necessary.

### 2.2. Spectroscopy Techniques

**Figure 3:**
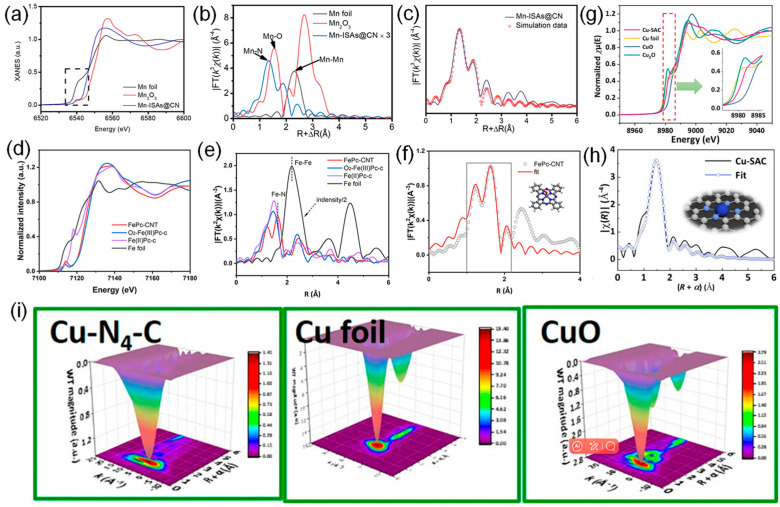
(**a**) Normalized Mn K-edge XANES spectra of the Mn foil, Mn_2_O_3_ and Mn-ISAs@CN. (**b**) Fourier transform (FT) k^3^-weighted χ(k)-function of the EXAFS spectra of the Mn K-edge of the Mn-ISAs@CN. (**c**) Corresponding Mn K-edge EXAFS fitting curve of Mn-ISAs@CN. Reproduced with permission [57]. Copyright 2020, Elsevier. (**d**) Normalized Fe K-edge XANES spectrum and (**e**) k^2^-weighted FT-EXAFS spectrum of FePc-CNT, together with those of commercial O_2_-Fe(III)Pc (O_2_-Fe(III)Pc-c), commercial Fe(II)Pc (Fe(II)Pc-c), and Fe foil standards for comparison. (**f**) Experimental and fitted EXAFS spectrum in R-space for the FePc-CNT catalyst (R range, 1–2.2 Å). Reproduced with permission [68]. Copyright 2023, American Chemical Society. (**g**) XANES analysis of Cu-SAC. (**h**) EXAFS of Cu-SAC. (**i**) WT contour plots of Cu-SAC. Reproduced with permission [69]. Copyright 2024, National Academy of Science, USA.

X-ray absorption spectroscopy (XAS) is a widely used technology to determine the electronic structure of catalysts, especially accurately probing the size, coordination environment, and chemical form of single atoms in SACs structure [54,59,60]. The XAS characterization is usually performed at the synchrotron radiation sources, which provide intense and tunable X-ray beams. X-ray absorption fine-structure spectroscopy (XAFS) is associated with the excitation process of core electrons of the X-ray absorbing atom [61]. XAS can be divided into three regions: pre-edge region, near-edge region (i.e., XANES), and post-edge region (i.e., EXAFS) [62,63,64,65]. XANES and EXAFS are the most commonly used XAS characterization techniques. The XANES region, which results from the excitation of core electrons to the valence and conduction bands, can provide information about the electronic valence state and characteristics of concerned elements [4,66]. While the EXAFS region, originating from the scattering interactions of photoelectrons with the neighboring atoms, can provide vital information on the identity of nearest-neighboring elements, coordination values, and interatomic bond distances [61,62,66]. To date, XAS gradually plays a pivotal role among various materials characterization techniques with the development of synchrotron radiation sources. For instance, monitored with XAS measurements, the dominant coordination mode of the Zn-N_4_ site in hollow porous carbon (HPC) derived from ZIF-8 was studied by Yang et al. [67]. XANES spectra illustrated that the average oxidation state of Zn species was probably situated between Zn^0^ and Zn^2+^ (close to Zn^2+^). Moreover, the fingerprinting signal peaks of Zn-Zn interactions were not observed in the profile of HPC-800 in EXAFS spectra, revealing the atomic dispersion of Zn in HPC-800. EXAFS spectra further verified that the Zn-N_4_ was the dominant coordination mode of Zn atoms in HPC-800. Analogously, Yang et al. found that Mn-ISAs@CN is between those of the Mn foil and the Mn_2_O_3_, implying that the atomically dispersed Mn species carried positive charges (Figure 3a). The EXAFS spectrum of Mn-ISAs@CN exhibits a stronger peak at about 1.3 Å, originating from the shell of Mn-N scattering, indicating the presence of an isolated single Mn atom (Figure 3b). The corresponding fitting results reveal that the first shell coordination number of the single Mn atoms in the Mn-ISAs@CN is four (Figure 3c). In addition, Zeng et al. found that the valence state of Fe in FePc-CNT was close to 3+ in XANES spectra (Figure 3d) [68]. The k^3^-weighted Fourier-transform spectra of the Fe K edge EXAFS further confirmed that single Fe atoms with a FeN_4_ configuration were incorporated into FePc-CNT (Figure 3e,f). Furthermore, the Cu K-edge XANES analysis of Cu-SAC showed that the Cu state of Cu-SAC was located between CuO and Cu_2_O (Figure 3g) [69]. The EXAFS showed that the coordination structure of Cu-SAC was based on Cu-N_4_-C configuration (Figure 3h). The monatomic dispersion of Cu species is further proved by the wavelet transform spectrum (Figure 3i). The advantages of XAS derive from the extremely high resolution and accuracy, and as a non-destructive characterization, it allows the acquisition of the required information without destroying the original structure of the catalyst. XAS can also be used for in situ testing to analyze changes in elemental valence and structure during catalytic reactions. However, to obtain high-quality XAS data, fine sample preparation and processing are required, including controlling factors, such as sample purity, morphology, and size. Furthermore, XAS is sensitive to experimental conditions (e.g., temperature, pressure, atmosphere, etc.), and therefore, experiments need to be performed to avoid these conditions interfering with the experimental results.

**Figure 4:**
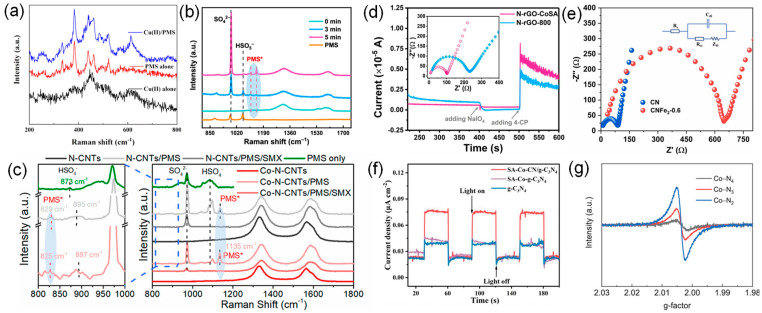
(**a**) Raman spectra of Cu(II), PMS, and Cu(II)/PMS in the absence of pollutants. Reproduced with permission [76]. Copyright 2020, American Chemical Society. (**b**) In situ Raman patterns, PMS* represents the active composites formed by combining PMS with catalysts. Reproduced with permission [72]. Copyright 2023, American Chemical Society. (**c**) In situ Raman spectra of different oxidation systems, PMS* represents the active composites formed by combining PMS with catalysts. Reproduced with permission [73]. Copyright 2021, American Chemical Society. (**d**) Current response after the sequential addition of IO_4_^−^ and 4-CP at the working electrode coated with the N-rGO-CoSA powder. The inset shows the EIS profiles of the N-rGO-CoSA and N-rGO-800 electrodes in the electrolyte solution. Reproduced with permission [80]. Copyright 2021, American Chemical Society. (**e**) Nyquist plots of CN and CNFe2-0.6-coated electrodes. Inset: equivalent fitting circuit. Reproduced with permission [83]. Copyright 2022, Elsevier. (**f**) Transient photocurrent responses of g-C_3_N_4_, SA-Co-g-C_3_N_4_, and SA-Co-CN/g-C_3_N_4_. Reproduced with permission [88]. Copyright 2022, John Wiley and Sons, USA. (**g**) Solid EPR spectra of Co-N_x_. Reproduced with permission [93]. Copyright 2022, John Wiley and Sons, USA.

The Raman spectrum is a useful tool to probe the structure of the SACs supported by carbon nanomaterials. Two distinguishable peaks in the Raman spectrum of the carbon materials are D band (1320–1355 cm^−1^) and G band (1570–1585 cm^−1^) [70]. The D band is related to the breathing mode of A_1g_ symmetry involving phonons near the K-zone boundary, which becomes active in the presence of defects/disorders. The G band is related to the E_2g_ stretching vibration mode of sp^2^ carbon, which is usually associated with sp^2^ sites in graphitic carbon. Thus, the intensity ratio of *I*_D_/*I*_G_ in a Raman spectrum is a common criterion to evaluate the defect and disorder degree of carbon materials or the ordered crystal structures of carbon atoms (the graphitization degree) [71]. Furthermore, in situ Raman tests are often used to observe adsorbed-state PMS (PMS*) and high-valent metal species [72,73,74,75]. Wang et al. showed that the new band near 612 cm^−1^ was strong evidence of the formation of Cu(III) in the Cu(II)/PMS system (Figure 4a), since this was a characteristic Cu-O stretching vibrational band for Cu(III) [76]. Adsorption of PMS is an important step in its being activated. As shown in Figure 4b,c, PMS* can be efficiently observed by an in situ Raman test [72,73].

Mössbauer spectroscopy is a spectroscopic method for the study of energy-level transitions in atomic nuclei. It is particularly suitable for catalysts containing elements such as iron, cobalt, and nickel. The Mössbauer spectroscopy technique is known for its ultrahigh-energy resolution, which makes it possible to achieve a precise characterization of the purity of catalysts when characterizing single-atom catalysts. The Mössbauer technique is not limited to the characterization of catalyst purity and coordination environment but also provides insight into the electronic state and coordination structure changes of the catalytic center during the reaction [77,78]. The development of in situ Mössbauer spectroscopy has further enhanced its application in the characterization of single-atom catalysts. The technique can probe the real structure and property changes of catalytic sites in real-time and quantitatively under reaction conditions, thus revealing the actual process of catalytic reactions.

Fourier-transform infrared spectroscopy (FTIR) of CO adsorption is also a commonly used method to determine whether single atoms are formed. Taking advantage of the specific interactions between CO molecules and atoms on the catalyst surface, the vibrational frequencies of CO molecules change when they are adsorbed on the surface of the catalyst, and these changes are manifested as specific absorption peaks in the FTIR spectra. By detecting and analyzing the positions, intensities, and shapes of these absorption peaks, the adsorption configurations of CO molecules on the catalyst surface can be deduced, and then it can be judged whether single-atom active sites have been formed on the catalyst surface. Because the atomic structure on the surface of SACs is different from that of conventional catalysts, the way and configuration of CO molecules adsorbed on the surface of the catalysts will also be different, and this difference can be clearly reflected in FTIR spectroscopy. Therefore, FTIR spectroscopy has become an effective means to characterize SACs. However, this means also has certain limitations, such as the possible simultaneous presence of other adsorbed species (e.g., water, oxygen, etc.) on the catalyst surface, which may interfere with the FTIR spectra. The limited sensitivity may make the technique challenging to detect SACs with low metal loadings.

### 2.3. Electrochemical Techniques

Electrochemical techniques, including chronoamperometric experiments, cyclic voltammetry (CV), and electrochemical impedance spectrum (EIS) measurements, can investigate the electrochemical properties, such as the reducibility and conductivity of SACs, which can be linked to redox potential, surface defects, electron transfer rate, etc. [79]. Chronoamperometric experiments are a commonly used method to prove the interaction of PMS/PDS with catalysts. The electron transfer could be identified from the increased electrochemical current on the working electrode. Long et al. found that the current response was monitored at glassy carbon electrodes (GCEs) loaded with N-rGO-CoSA and N-rGO-800 after successive injections of IO_4_^−^ and 4-CP (Figure 4a) [80]. After the injection of IO_4_^−^, the current on the electrodes covered by the two catalysts decreased significantly, which was caused by the electron transfer process after the formation of the complex between the catalyst and IO_4_^−^. The observed increase in current after the addition of 4-CP is attributed to the electron transfer process from the electron donor (4-CP) to the acceptor (complex). Therefore, the above results suggest that both N-rGO-CoSA and N-rGO-800 can act as electron shuttles to facilitate the electron transfer process from 4-CP to IO_4_^−^, leading to the nonradical oxidation of 4-CP. The increase in the current intensity of N-rGO-CoSA was more significant than that of N-rGO-800, indicating that the introduction of Co single atomic site greatly enhanced the catalytic performance. An EIS measurement can be applied to assess the electrical conductivity and electron transfer ability of catalysts [13,81,82]. It is well known that the smaller arc radius or semicircular radius of Nyquist plots indicates a lower charge-transfer resistance. The EIS of N-rGO-CoSA and N-rGO-800 shows that the electron transfer resistance (Ret) of the former is smaller than that of the latter. Therefore, the Co single atomic site endowed the catalyst carrier with higher conductivity and better electron transfer mediating ability, accelerating the kinetic process of 4-CP oxidation. Zhu et al. measured electrochemical impedance using a Nyquist diagram and equivalent fitting circuit [83] and found that the charge-transfer resistance (Rct) of CNFe2-0.6 was 566.80 Ω, much higher than that of CN (80.90 Ω) (Figure 4b). Since CN is a semiconductor, a higher CNFe2-0.6 Rct indicates poor conductivity, which is speculated to be due to the abundant nanopore disturbing the ordered electron flow. Furthermore, it is analyzed that the electron transfer mechanism dominates the degradation of pollutants. Electrochemical testing allows real-time measurements and monitoring of changes in catalyst performance during catalytic reactions, facilitating the understanding of catalyst deactivation mechanisms. Electrochemical techniques also typically have a high sensitivity for detecting minor current or voltage changes, making them uniquely suited for evaluating low-loading or high-activity SACs. However, the electrochemical testing process may be interfered with by a variety of factors, such as electrode materials, electrolyte composition, and test temperature. These interfering factors may affect the accuracy and reliability of the test results.

### 2.4. Other Characterization Techniques

Apart from the techniques discussed above, some other characterization approaches also have been widely employed to explore the structure and properties of SACs applied in SR-AOPs. N_2_ adsorption and desorption isotherm is the most typical physical adsorption process, which is an effective way to characterize the pore size distribution, pore volume, and surface area of SACs. The characters of pores (micropore, mesopore, and macropore) in SAC materials can be identified according to the type of isotherm (I–VI) and the possible type of hysteresis loop (H1-H5) in N_2_ adsorption and desorption isotherm. Additionally, the surface area and pore volume of SACs can be calculated via the corresponding calculation model, such as Langmuir and Brunauer–Emmett–Teller (BET), according to International Union of Pure and Applied Chemistry (IUPAC) classification. It is remarkable that, in almost all cases, the introduction of metal species will cause a decrease in the surface area of SACs to some extent, considering the mass occupation of metal species [54]. Likewise, the surface area of SACs presents an apparent decrease because the frameworks of the precursor tend to shrink, and many pores completely disappear after long-time harsh heat treatment [84]. Thermogravimetry (TG) is a powerful tool that can be used to characterize the thermostability and constituent of the materials via estimates of the correlation between quality and temperature of the catalyst at a specific temperature. Transient photocurrent tests of photoelectrochemical measurements are used to study the electron–hole-pair separation and carrier migration characteristics of photocatalysts and to evaluate the photocatalytic performance of the catalysts [81,85,86,87]. Qian et al. found that the photocurrent density of pure g-C_3_N_4_ and SA-Co-C_3_N_4_ is almost the same [88], but much smaller than that of SA-Co-CN/g-C_3_N_4_, indicating that the heterogeneous structure promotes the generation and transport of carriers, and the construction of SA-Co-CN/g-C_3_N_4_ heterogeneous structure can improve the separation and migration of carriers (Figure 4c). Electron paramagnetic resonance (EPR) spectroscopy is a direct and advanced technique for detecting oxygen vacancy and carbon material-defect levels [13,89]. It provides information about unpaired electrons on the surface of the material as a signal of oxygen vacancies and defects [90,91,92]. EPR spectroscopy is based on paramagnetic samples (with an unpaired electron) that, in a suitable magnetic field, can absorb electromagnetic radiation [86]. Liang et al. use solid-state EPR spectroscopy (Figure 4d), proving that nitrogen vacancy is formed during the construction of the Co-N_2_ coordination structure [93]. Co-N_4_ shows a slight paramagnetic signal with a g-value of about 2, which can be assigned to unpaired electrons, indicating the formation of a small number of nitrogen vacancies. The corresponding peak intensity of EPR in Co-N_3_ and Co-N_2_ increased significantly to a certain extent, respectively, indicating a large number of nitrogen vacancies in Co-N_2_ samples. In summary, there are various techniques for the characterizations of SACs, and each technique has its unique advantages and limitations. In practical applications, it is necessary to choose the appropriate characterizations technique or a combination of techniques according to the research purpose and sample characteristics in order to obtain comprehensive and accurate information.

## 3. Theoretical Calculation

In addition to EXAFS and HAADF-STEM commonly used in SACs characterization, density functional theory (DFT) calculation is a powerful weapon to verify the presence state, reaction path, and specificity of catalysts [38,46,94]. Density functional theory is a quantum mechanical approach that studies the electronic structure of multi-electron systems. So, density functional theory has a wide range of applications in physics and chemistry, especially in studying molecular and condensed matter properties. The main focus in the electronic structure calculations is on the ground-state energy and wave function of the electrons, implying an atomic mean kinetic energy of 0, i.e., a system temperature of 0 K [95,96]. Such a setup simplifies the calculations and allows us to focus on the quantum-mechanical behavior of the electrons without having to take into account the effect of the temperature on the electron distribution [44,97]. A comparison of DFT calculations with experimental results at room temperature is a critical step in verifying the accuracy of theoretical predictions. Classical methods of electron structure theory, such as the Hartree–Fock method, are based on complex multi-electron wave functions [98,99]. The main goal of density functional theory is to replace the wave function with electron density as a fundamental quantity. The material science calculations of the DFT are often combined with related experiments as a supplement and extension to the experimental discipline. The mechanism behind the matter can be further explored by studying the structure of materials (e.g., bond length and vibration).

Applications of theoretical calculation in the field of SACs include studying the monatomic dispersion process, stability and electronic properties after loading, and catalysis processes [18,86,100,101]. In single-atom catalysis, the content that can be calculated mainly includes adsorption energy, state density, differential charge analysis, and catalytic reaction energy barrier. Among them, the adsorption energy (*E*_ads_) calculation reflects the capture ability of monatomic materials to reactants; the state density result reflects the electronic structure of materials; the difference charge analysis reflects the electron transfer between SACs materials and reactants; and the reaction energy barrier (Δ*G*) calculation can show that the energy change in the catalytic process demonstrates the activity and selectivity of catalysts [43,82,102,103]. Although SACs have received widespread attention, the changes in SACs in the catalytic reaction and the related mechanism of catalytic reaction have not been deeply understood. In addition, people still lack theoretical means to investigate the stability of single atomic sites in the reaction process, except for experimental observation. It is of great significance for designing and developing highly active SACs to analyze the stability of monatomic catalysts and the changes in small molecules and substrates in the catalytic reaction process utilizing DFT calculation.

**Figure 5:**
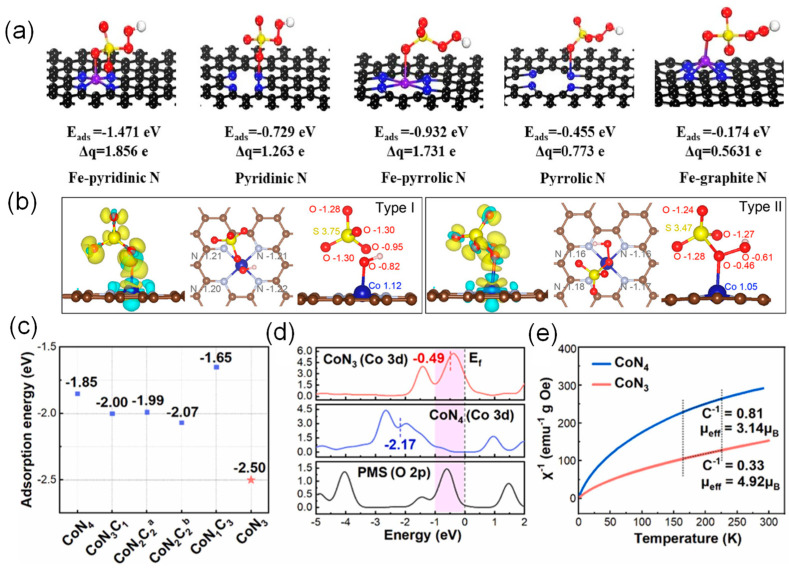
(**a**) Adsorption energy on different FeN_x_–C and N–C sites and the corresponding O–O bond length in PMS and Bader charge after PMS adsorption. Reproduced with permission [108]. Copyright 2022, American Chemical Society. (**b**) Charge-density differences of PMS adsorbed onto Co-TPML via the coordination of O atom adjacent to the H atom (Type I) and O atom adjacent to the S atom (Type II) in the PMS bond. Yellow denotes the electron accumulation, and cyan represents the electron depletion. Reproduced with permission [109]. Copyright 2021, American Chemical Society. (**c**) Adsorption energy profiles of PMS toward different Co-N-C configuration models. (**d**) Projected density of states (PDOS) of Co 3d in CoN_3_, Co 3d in CoN_4,_ and adsorbed O 2p in PMS. (**e**) Temperature-dependent inverse susceptibilities over CoN_3_ and CoN_4_ (C refers to Curie constant). Reproduced with permission [110]. Copyright 2023, Elsevier.

Radicals (^•^OH and SO_4_^•**–**^) and nonradicals species (catalyst-mediated electron transfer mechanism, high-valent metal, and ^1^O_2_) are usually present in reactive oxygen species (ROS) in PMS-based SAC catalysts [49,104,105,106,107]. The adsorption and subsequent bond breaking of PMS molecules can be analyzed by theoretical calculations, and the reaction mechanism can be further explained. Zhang et al. performed DFT calculations to explore PMS adsorption on the possible catalytic sites [108]. Figure 5a shows that the adsorption energies on Fe–pyridinic N_4_–C, pyridinic N–C, Fe–pyrrolic N_4_–C, pyrrolic N–C, and Fe–graphitic N_4_–C were calculated to be −1.471, −0.729, −0.932, −0.455, and −0.174 eV, respectively. The O–O bond lengths (*l*_O–O_) in PMS were 1.562, 1.438, and 1.490 Å after adsorbing on the Fe–pyridinic N_4_–C, Fe–pyrrolic N_4_–C, and Fe–graphitic N_4_–C, respectively, which were elongated compared with original PMS molecules (1.410 Å). The results indicate that Fe–pyridinic N_4_–C interacts more strongly with HSO_5_^−^ compared with other configuration models due to the more considerable adsorption energy and longer O–O bond length. Chu et al. demonstrated the critical role of the transfer of delocalized π-electrons in tetrapyridomacrocyclic (TPML) in facilitating O–O bond cleavage and stabilizing the surface-bound ^•^OH [109]. The difference-charge and Bader-charge analyses were performed on two possible adsorption configurations: coordinating the Co atom with the O atom adjacent to the H atom (Type I adsorption) or the O atom adjacent to the S atom (Type II adsorption) of O–O bond (Figure 5b). A strong charge transfer from Co-TPML to PMS occurs via Type I adsorption configuration, elongating the O–O bond to 2.01 Å and enabling spontaneous dissociation of O–O bond to surface-bound ^•^OH and free SO_4_^•–^ species. In contrast to Type I adsorption, while strong adsorption of PMS on Co via Type II configuration also occurs, the charge transfer is minor, and the O–O bond of PMS is slightly elongated from 1.44 to 1.46 Å, indicating that the PMS–Co interaction via Type II adsorption configuration is insufficient for direct O–O bond dissociation. In addition, Song et al. designed unsaturated coordination of a single-atom Co-N_3_ catalyst in contrast to a conventional Co-N_4_ catalyst for the activation of PMS [110]. The DFT results showed that the unsaturated coordination model containing N vacancies (Co-N_3_) has more vital adsorption energy compared to the saturated coordination model (Co-N_4_, Co-N_3_C_1_, Co-N_2_C_2_, and Co-N_1_C_3_) (Figure 5c). The higher overlap between Co 3d and O 2p near the Fermi energy level, the narrower gap between the d-band center and the Fermi level, and the higher spin state indicated the strong interaction between the Co center and PMS and the enhanced adsorption capacity of the active center (Figure 5d,e).

**Figure 6:**
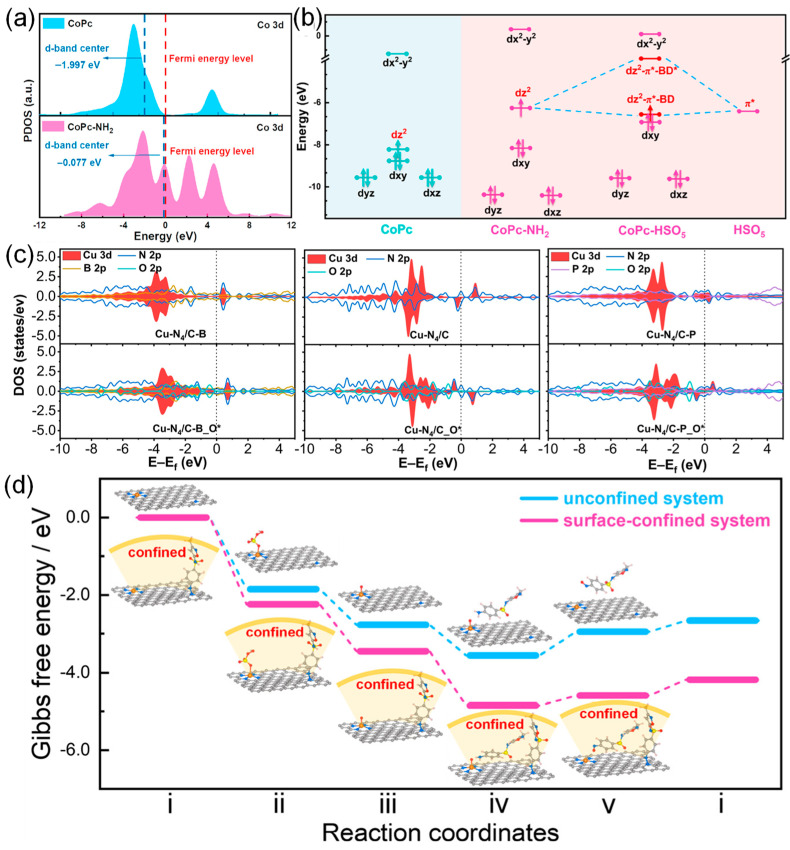
(**a**) PDOS of Co 3d in CoPc and CoPc-NH_2_. (**b**) Energy levels of Co 3d orbital and orbital interactions between PMS and CoPc-NH_2_. (**c**) Reaction pathways of PMS activation at Co–N_4_ sites and corresponding Gibbs free energy (eV). Reproduced with permission [72]. Copyright 2023, American Chemical Society. (**c**) PDOS of Cu atom, heteroatoms in the substrate, and oxygen of PMS adsorbed on the Cu center (EF is marked in each graph with the black dashed line). Reproduced with permission [111]. Copyright 2022, National Academy of Science. (**d**) Reaction pathways of PMS activation at Fe–N_4_ sites and the corresponding Gibbs free energy (eV). Reproduced with permission [113]. Copyright 2023, American Chemical Society.

Recently, our group employed DFT to illustrate the effects of model catalysts with different electronic structures on the activation of PMS [72]. As displayed in Figure 6a, compared to CoPc/G, the Co 3d d-band center in CoPc/G-NH_2_ shifted positively from −1.997 eV to −0.077 eV, which led to a decreased occupancy and enhanced the adsorption strength of PMS. Additionally, the closer proximity of the d-band center to the Fermi level accelerated charge transfer at the reaction interface, thereby lowering both the free energy barrier and activation energy. Moreover, as shown in Figure 6b, the molecular orbitals analysis revealed that PMS activation was predominantly governed by the 3dz^2^ orbital in the Co(II) center and π* from the HSO_5_ group. The amino ligand modification notably enhanced the dz^2^ orbital compared to CoPc, which rendered CoPc-NH_2_ more prone to electron donation, consequently facilitating PMS activation. In addition, Zhou et al. integrate electron-deficient boron (B) or electron-rich phosphorus (P) heteroatoms into carbon substrates to tune the electronic structure of Cu-N_4_ sites for PMS activation [111]. As shown in Figure 6c, the partial density of states (PDOS) for the Cu centers, heteroatoms in the substrate, and oxygen of PMS adsorbed on the Cu sites were calculated. In apparent contrast to primary Cu-N_4_/C, the PDOS of Cu sites in Cu-N_4_/C-B displays a negative shift, indicating a decrease in the d-band center. Comparatively, the PDOS of Cu atoms in Cu-N_4_/C-P moves toward the opposite direction, with an increased d-band center. Additionally, the PDOS for PMS adsorbed on Cu centers shows that Cu-N_4_/C-B has a relatively weak interaction with PMS compared to Cu-N_4_/C. Therefore, heteroatom functionalization can effectively optimize the electronic structure of Cu-N_4_ sites, boosting the activation of PMS. Furthermore, DFT can also be used to calculate the Gibbs free energy of PMS activation from a thermodynamic point of view [112,113]. FeN_4_ structures were constructed to obtain single-atom catalysts for generating FeN_4_=O intermediate [113]. The corresponding energy profiles (surface-confined and unconfined systems) were performed to probe the insights of PMS activation and SMX oxidization mechanism on the FeN_4_ site (Figure 6d). The results showed that the total energy released at the FeN_4_ site during the reaction over the surface-confined system is much larger than that of the unconfined system, suggesting that the oxidation of SMX is thermodynamically favorable.

## 4. Conclusions and Perspectives

In this work, the advanced characterization methods and preparation strategies of SACs are reviewed, and the catalytic applications of SACs in Fenton-like chemistry and the application directions of theoretical calculation in single atomic catalysis are introduced. Although SACs have made significant progress in many fields, some research directions still need to solve some problems. There is still a long way to go before industrial application. At present, the following challenges need to be solved:

(1) The changes in SACs in the catalytic reaction process and the related catalytic reaction mechanism still need to be further understood by employing theoretical calculations.
(2) The stability of the single atomic site of SACs in the reaction process needs further improvement.
(3) Under working conditions, direct knowledge of the dynamic changes in active sites during catalytic reactions is still limited. Real-time monitoring of real active sites by in situ measurements, such as in situ HAADF-STEM, XAS, and diffuse infrared Fourier-transform spectroscopy (DRIFTS), helps to clarify the catalytic mechanism.
(4) It is still necessary to regulate the electronic structure of the single atomic active site through local electronic structure engineering to improve the intrinsic catalytic activity of the single atomic site and improve the catalytic reaction kinetics.
(5) More efforts should be focused on the practical industrial application of SAC, and more attention should be paid to technical issues, reactor size, facility capacity, and reaction economics.

## Data Availability

The original contributions presented in the study are included in the article; further inquiries can be directed to the corresponding authors.
